# Investigation of β-Sitosterol and Prangol Extracted from *Achillea Tenoifolia* Along with Whole Root Extract on Isolated Rat Pancreatic Islets

**Published:** 2018

**Authors:** Mahban Rahimifard, Azadeh Manayi, Maryam Baeeri, Mahdi Gholami, Soodabeh Saeidnia, Mohammad Abdollahi

**Affiliations:** a *Toxicology and Diseases Group,* *Pharmaceutical Sciences Research Center, Tehran University of Medical Sciences, Tehran 1417614411, Iran. *; b *Medicinal Plants Research Center, Tehran University of Medical Sciences, Tehran 1417614411, Iran.*; c *Department of Toxicology and Pharmacology, Faculty of Pharmacy, Tehran University of Medical Science, Tehran, Iran.*; 1 M.R. and A.M. contributed equally to this work.

**Keywords:** *Achillea tenuifolia* root, Prangol, β-Sitosterol, Pancreatic islet cells, Oxidative stress

## Abstract

The genus *Achillea* (Asteraceae) consisting of important medicinal species, growing wildly in Iran, of which *A. tenuifolia* is found in Iran-o-Turan regions. Regarding the traditional use of *Achillea* species for treatment of diabetes and also lack of information on phyto-constituents of *A. tenuifolia* underground parts, in this study anti-diabetic activity of the plant have been reported. In order to find the main active components, underground parts of the plant were extracted with water and fractioned by hexane, ethyl acetate, and methanol and the separation of the main compounds were carried out *via* medium pressure liquid chromatography (MPLC). Also, anti-diabetic effects of the extract were investigated on rat pancreatic islets. The root extract of the plant as well as the compound β-sitosterol showed moderate α-amylase inhibitory activity, however prangol did not suppress the enzyme activity. The results of islet cells’ bio-function assays revealed that the herb root extract was able to increase the secretion of insulin in high concentration (10 mg/mL) and improved the cell viability with no toxicity in all doses. Furthermore, the herbal extract could reduce the levels of reactive oxygen species (ROS) and lipid peroxidation (LPO). The plant extract also significantly decreased the enzyme activity for both caspase-3 and -9 and increased the antioxidant capacity of the isolated cells. Taking together, preparations or extracts from the underground parts of the plant are good candidates for further anti-diabetic investigation and clinical trials.

## Introduction

Diabetes mellitus (DM) is one of the metabolic diseases representing by symptoms of high blood glucose levels over a prolonged period, resulting in frequent urination, increased thirst, and increased hunger (1). Generally in diabetes, the pancreas cannot produce enough insulin, or the cells of the body may not respond properly to the insulin produced. There are three main types of DM including “Type 1 DM” (insulin-dependent diabetes mellitus (IDDM) or juvenile diabetes) related to the pancreatic cells’ failure to produce enough insulin; “Type 2 DM” which occures due to insulin resistance of body cells and so-called "non-insulin-dependent diabetes mellitus" (NIDDM) or "adult-onset diabetes" that may be results of obesity and not enough exercise; gestational diabetes is the third type and occurs in pregnant women without a previous history of diabetes (2). Management of DM focuses on keeping blood sugar levels as close to normal as possible, without causing hypoglycemia (3). Type 1 DM is typically treated with a combination of regular and NPH insulin, or synthetic insulin analogs, while a long-acting formulation is usually added for type 2 DM. However, metformin is generally recommended as a first line treatment for type 2 DM (4, 5). 

Obviously, diabetes is a major systemic disease affecting a significant proportion of the population worldwide (6). Regarding the point that “α-amylase enzyme” plays a critical role in digestion of carbohydrates, the inhibitors of α-amylase (AAI) are important in the treatment of diabetes, obesity, and periodontal diseases. Medicinal plants are the natural sources of AAI, which are used as therapeutic or functional food resources. A bibliography shows that many sorts of phytochemicals and plant species have been reported as the α-amylase inhibitors, especially whose constituents belong to the phenolic compounds (7). Furthermore, Persian traditional medicine introduced promising candidates for the treatment of diabetes (8). For this reason, scientists in related fields are currently concentrating on finding the anti-diabetic constituents from natural origin (9). 

*Achillea* (Asteraceae) species are well-known medicinal plants with therapeutic applications worldwide. Some of these species have been reported for their anti-diabetic activity. For instance, the effect of *A. santolina* on blood glucose level, serum nitric oxide (NO) concentration and the oxidative stress in rat pancreatic tissue have been reported. Reducing blood glucose level, serum NO, pancreatic malonedialdehyde, protein carbonyls, and advanced oxidation protein products levels are found as the major results of treatment with this plant. The authors concluded that the content of GSH (reduced glutathione) was also restored to the normal level of the control group. Regarding the enhancement of antioxidant enzymes by the plant, the authors suggested that the hypoglycemic activity could be attributed to the antioxidative potential of the plant (10). Here in the present study, we focused on *A. tenuifolia, *growing wildly in Iran, which has previously been investigated for its antioxidant potential (11), total phenol content and micro-morphological characterizations (12). Due to the high potential of antioxidant activity and total phenol constituents of the plant roots, we employed root extract for more investigations on its anti-diabetic activity of the islets bio-function isolated from rat pancreas. Additionally, the major phyto-constituents of the root extract and their α-amylase inhibitory activity have been investigated as well. 

## Experimental

*Plant material and extraction*

The roots of *A. tenuifolia* were collected from Qazvin province in June (2011), and identified by Dr. Yousef Ajani. A herbarium specimen (No. 1624) has been deposited in the Herbarium of Institute of Medicinal Plants, Jahade-Daneshgahi (ACECR), Karaj, Iran. The plant root was cleaned and dried in the shade under room temperature. The dried root powder (700 g) was percolated by distilled water three times for 72 h, and then the resulted extract was concentrated by rotary evaporator and dried in freeze dryer (15 g). The extract was used for further pharmacological investigation.

*Isolation process of the main compounds by medium pressure liquid chromatography (MPLC)*

The aqueous extract was further fractionated by hexane, ethyl acetate, and methanol solvents. The fractions obtained by washing were concentrated again using a rotary evaporator, and consequently dried in freeze dryer resulting hexane (0.8 g), ethyl acetate (1.9 g), and methanol fractions. The hexane fraction was submitted to a silica gel column on the MPLC chromatograph. The column was eluted by hexane-chloroform (5:5-8:2; flow rate: 2.5 mL/min; UV detector at 254 nm) to gain three main sub-fractions (A-C). The sub-fraction B (0.4 g) was subjected to silica gel column chromatography with a solvent system as hexane-ethyl acetate (19:1, 8:2) to give two compounds **1** and **2**. The obtained compounds were then submitted to a sephadex LH_20_ CC and eluted with chloroform: methanol (5:5) to gain the pure compounds **1** (6 mg) and **2 **(2.1 mg). 

ß-Sitosterol [1]: ^1^H-NMR: (500 MHz, CDCl_3_): δH 0.68 (3H, s, H-18), 0.81 (3H, br s, H-26), 0.82 (3H, br s, H-27), 0.84 (3H, br s, H-24b), 0.92 (3H, d, *J *= 6.7 Hz, H-21), 1.01 (3H, s, H-19), 3.52 (1H, m, H-3), 5.35 (1H, m, H-6). ^13^C-NMR (125 MHz, CDCl_3_): δC (from C-1 to C-27) 37.3, 31.7, 71.8, 42.3, 140.8, 121.7, 31.9, 31.9, 50.2, 36.5, 21.1, 39.8, 42.3, 56.8, 24.3, 28.3, 56.1, 11.9, 19.8, 36.2, 18.8, 34.0, 26.1, 45.8, 29.2, 19.0, 19.4, 23.1 (C-24a), 12.0 (C-24b).

Prangol [2]: ^1^H (500 MHz, CDCl_3_) and ^13^C- NMR (125 MHz, CDCl_3_) of this compound are shown in Table 1.

*α-Amylase inhibitory activity*

The α-amylase inhibition assay was performed by some modification in the method proposed by Giancarlo *et al.* (13). Fifty microliter of each plant extracts and 150 µL of starch solution as well as 10 µL of enzyme were mixed in a 96 well plate and incubated at 37 °C for 30 min. Subsequently, 20 µL of sodium hydroxide and 20 µL of color reagent were added and the closed plate placed into a 100 °C water bath. After 20 min, the reaction mixture was removed from the water bath and cooled, thereafter α-amylase activity was determined by measuring the absorbance of the mixture at 540 nm in Elisa stat fax 2100 (Awarness Technology Inc). The inhibition percentage of α-amylase was assessed by the following formula:

I α –Amylase %= 100× (ΔA _control _– ΔA _sample_) /ΔA _control_

ΔA _control_=A _control test_-A _control blank_

ΔA _sample_= A_ sample test_-A _sample blank_

Statistical analysis was performed using the StatsDirect and Probit analysis. The results of this biological assay are summarized in Table 2.

*Isolation and incubation of islets*

The islets were isolated from male adult Wistar rats (2-3 mouths; weight ~250 ± 25 g). All animal care was performed according to the animal welfare Act approved by Pharmaceutical Sciences Research Center Ethics Committee with code number of 90-04-151-16052. After they were anesthetized by injection of sodium pentobarbital (60 mg/kg), the pancreas was inflated by injecting of Krebs buffer into duodenal duct. Inflated pancreas was removed and cut into small pieces. collagenase enzyme was then added to pancreas at 37 °C for 10 min. All the tissues surrounding islets and fat were removed. Under the stereo microscope, islets of the similar size were isolated by hand picking using a sampler (14). Various concentrations of the plant extract (1, 10, 100, 1000 and 10000 μg/mL) were dissolved in RPMI medium culture and exposed to the islets for 24 h at 37 °C with 0.5% CO_2_.

*Cell viability assay*

Cell viability was measured by dimethylthiazol-2-y1-2,5-diphenyltetrazolium bromide (MTT) assay. After 24 h incubation with various doses of the plant extract, the medium was removed and 20 μL of MTT (0.5 mg/mL) was added and incubated for 3 h at 37 °C. The violet crystal was dissolved in dimethyl sulfoxide (DMSO) and remained for 30 min, and then the optical density was measured at 570 nm using an ELISA reader.

**Table 1:** ^1^H (500 MHz, CDCl_3_) and ^13^C- NMR (125 MHz, CDCl3) of the compound **1** (prangol).

**Carbon**	^13^ **C-NMR**	^1^ **H-NMR**
**2**	161.3	-
**3**	112.8	6.22 (d, *J*= 9.5 Hz, 1H)
**4**	139.2	8.14 (d, *J*= 9.5 Hz, 1H)
**5**	148.5	-
**6**	114.1	-
**7**	158.0	-
**8**	94.6	7.08 (s, 1H)
**4a**	107.1	-
**8a**	152.4	-
**1'**	-	-
**2'**	145.2	7.58 (d, *J*= 2.5 Hz, 1H)
**3'**	104.8	6.97 (d, *J*= 2.5 Hz, 1H)
**4'**	-	-
**5'**	-	-
**1"**	71.7	4.43 (dd, *J*= 9.5, 7.5 Hz, 1H) 4.54 (dd,* J*= 9.5, 3 Hz, 1H)
**2"**	74.4	3.90 (dd, *J*= 7.5, 3 Hz, 1H)
**3"**	76.5	-
**4"**	26.6	1.35 (s, 3H)
**5"**	25.1	1.30 (s, 3H)

**Table 2.** Percent of α-Amylase inhibitory activities (% AAI) and IC_50_ values of the extract and active constituents of *A. tenuifolia*

**Extract or Compounds**	**Conc. (mg/mL)**	**% AAI** ** (Mean ± SD)**	**IC**_50 _ **(mg/mL)** *
***tenuifolia*** **hexane extract**	10	50.2 ± 1.0	10.4 ± 1.6
	15	34.1 ± 1.3	
	20	10.8 ± 1.8	
	25	6.8 ± 1.9	
**Acarbose**	10	67.3 ± 2.1	6.6 ± 2.1
	15	69.2 ± 3.9	
	20	73.8 ± 1.9	
	25	82.1 ± 2.3	
**β-Sitosterol**	10	25.5 ± 0.02	18.9 ± 2.1
	15	28.7 ± 0.03	
	20	57.4 ± 0.02	
	25	59.4 ± 0.03	
**Prangol**	10	6.0 ± 0.02	> 200**
	15	6.3 ± 0.02	
	20	7.2 ± 0.01	
	25	7.4 ± 0.03	

Conc: Concentration, % AAI: percent of α-Amylase inhibitory activities);

* IC50 value is the concentration of sample required for 50% inhibition. Each value is expressed as mean ± SD (n = 3);

** The IC50 value more than 200 shows almost no activity.

**Figure 1:**
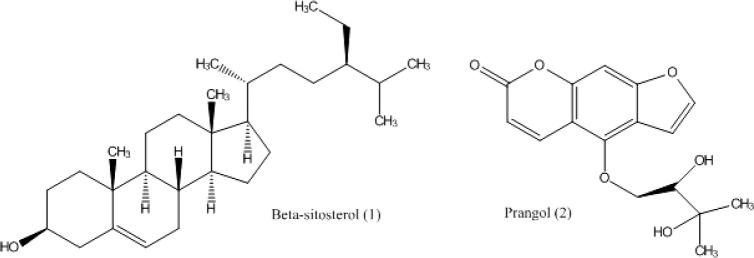
Chemical structures of the isolated compounds from *A. tenuifolia.*

**Figure 2:**
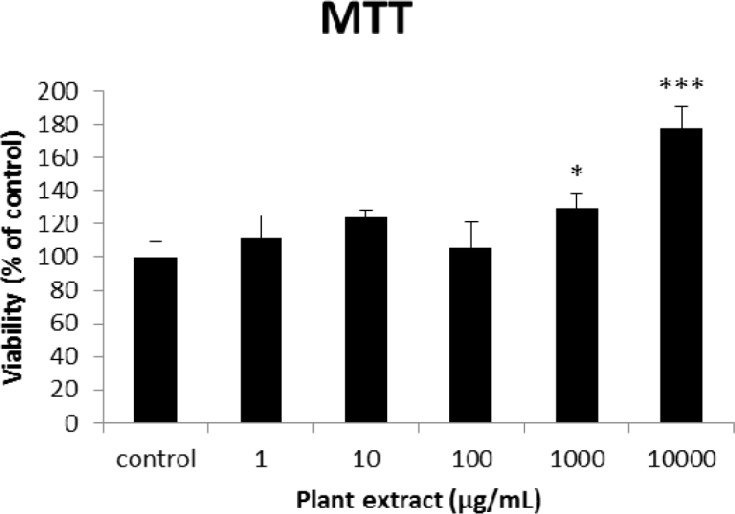
The results of MTT assay on different concentrations of the root extract of *A. tenuifolia. *Each group contained 10 rats’ pancreatic islets; * and *** mean significant increase of mitochondrial activity compared to the control group by *p* value < 0.05 and *p* value < 0.001; Control group contains islets that were not treated by extract

**Figure 3:**
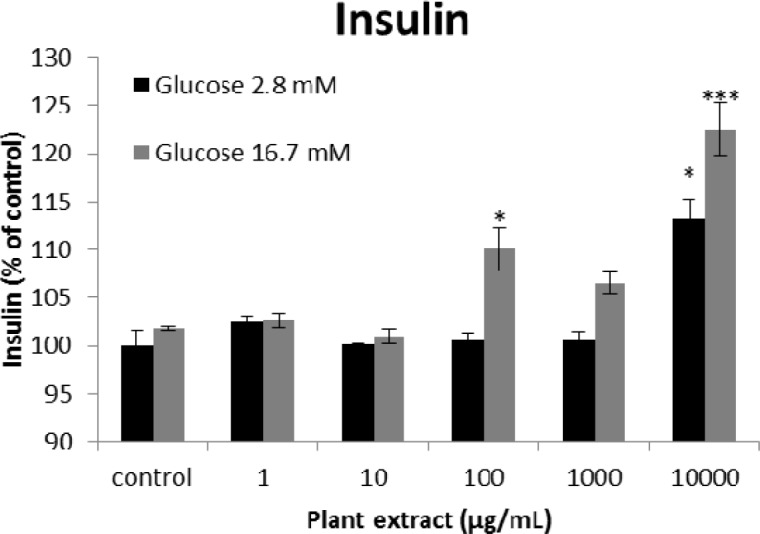
The results of insulin secretion from different groups of rats’ pancreatic islets treated with different concentrations of the root extract of *A. tenuifolia. *Each group contained two different sub-groups of 10 rats’ pancreatic islets that stimulated with 2.8 and 16.7 mM of glucose; * and *** mean significant increase of secretion compared to the control group by *p* value < 0.05 and *p* value < 0.001; Control group contains islets that were not treated by extract

**Figure 4.**
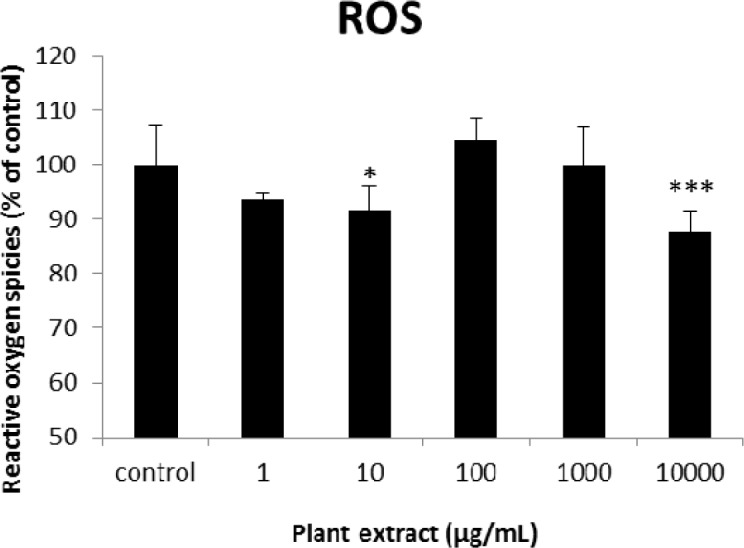
The results of cellular reactive oxygen species (ROS) measurement for groups of rats’ pancreatic islets treated with different concentrations of the root extract of A. tenuifolia. * and *** mean significant decrease of ROS generation percentage compared to the control group by *p* value < 0.05 and *p* value < 0.001; Control group contains islets that were not treated by extract

**Figure 5:**
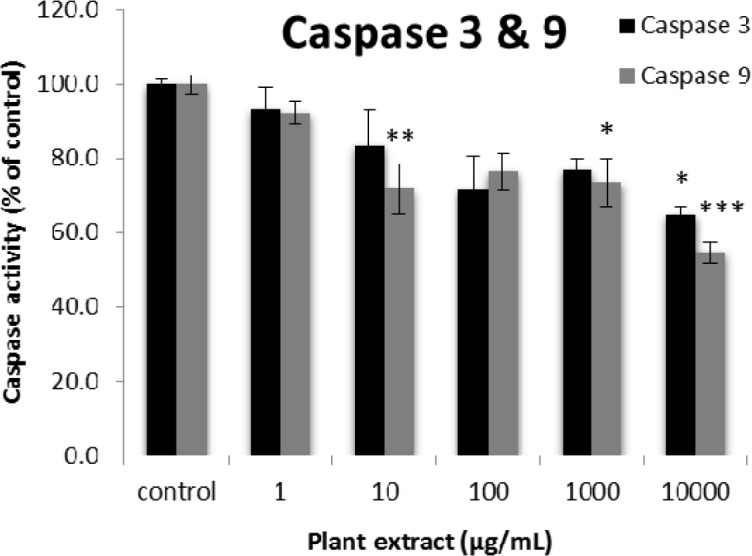
The results of caspase-3 and -9 activity percentages for groups of rats’ pancreatic islets treated with different concentrations of the root extract of A. tenuifolia. *, ** and *** mean significant decrease of caspase activity percentage compared to the control group by *p* value < 0.05, *p* value < 0.01 and *p* value < 0.001, respectively; Control group contains islets that were not treated by extract

**Figure 6:**
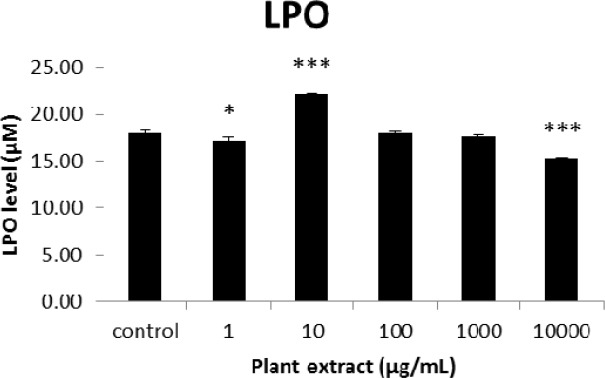
The results of cellular lipid peroxidation (LPO) measurement for groups of rats’ pancreatic islets treated with different concentrations of the root extract of A. tenuifolia. * and *** mean significant changing of LPO percentage compared to the control group by *p* value < 0.05 and *p* value < 0.001; Control group contains islets that were not treated by extract

**Figure 7:**
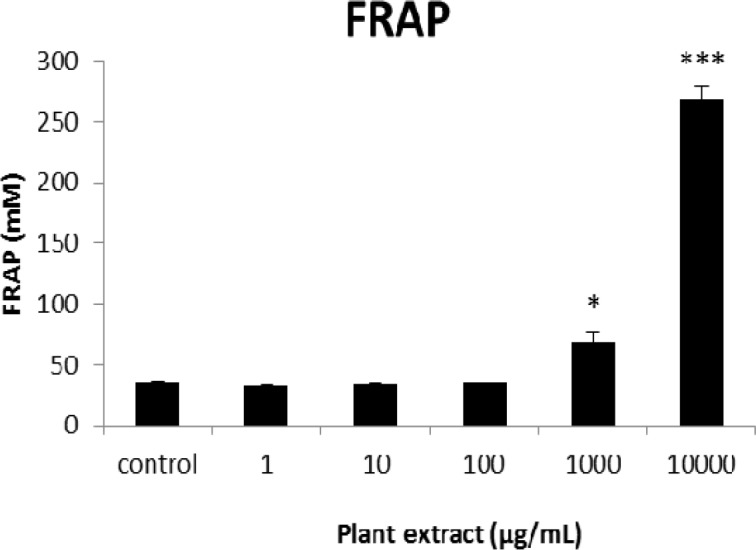
The results of FRAP assay for groups of rats’ pancreatic islets treated with different concentrations of the root extract of A. tenuifolia. * and *** mean significant increase of antioxidant capacity of cells compared to the control group by *p* value < 0.05 and *p* value < 0.001; Control group contains islets that were not treated by extract

*Insulin secretion*

The function of islets was assessed by insulin secretion in glucose static incubation. After exposing the islets with different concentrations of the plant extract for 24 h, the media and extract were removed from islets by washing twice with Krebs-HEPES buffer. Then, the islets were incubated with 2.8 mM glucose for 30 min. After that, islets were divided into two groups, the first were treated by 2.8 mM glucose (the basal dose) and the second by 16.7 mM glucose (stimulant dose) for 30 min at 37 °C. Islets were centrifuged and insulin assay in the supernatant was performed using rat insulin ELISA kit (15).

*Intracellular reactive oxygen species (ROS)*

ROS were measured using 2ꞌ,7ꞌ-dichlorodihydrofluorescein diacetate (DCFH-DA). Islets were collected and washed three times and homogenized by extraction buffer. Afterwards, they were centrifuged and 10 μM of 2**ꞌ**,7ꞌ-dichlorodihydrofluorescein (DCFH) was added to 162 μM supernatant. After 30 min incubation in 37 °C, the ELISA fluorimeter was used to measure absorbance every 1 min up to 60 min (16). 

*Caspase activity analysis*

Caspase-3 and -9 activities were determined by a colorimetric assay according to the manufacturerꞌs protocol. Briefly, cells were lysed in the supplied lysis buffer and were incubated 4 h with the supplied reaction buffer containing dithiothreitol and AC-DEVD-pNA (specific for caspase-3) and Ac-LEHD-pNA (specific for caspase-9) as substrates (Alexis Biochemicals Co.) at 37 °C. The reaction was measured by changes in absorbance at 405 nm using an ELISA reader. Enzyme activity was expressed as the fold increase in the proportion of apoptotic cells over that of non-treated control cells (17).

*Ferric reducing antioxidant power* (*FRAP) assay*

The key solutions for performing FRAP assay were prepared as follows: a) Acetate buffer 300 mM, pH 3.6; b) 2,4,6-tripyridyl-striazine (TPTZ): 10 mM in 40 mM HCl; c) FeCl_3_. 6H_2_O:20 mM. The FRAP solution was prepared by mixing a, b and c in the ratio of 10:1:1 just before testing. The standard was FeSO_4_.7H_2_O: 0.1-1.5 mM in methanol. FRAP solution was mixed with 0.4 mL distilled water and certain concentration of the plant extract (80 μL) and incubated at 37 °C for 10 min. The absorbance of the reaction mixture was measured at 593 nm (18).

*Lipid peroxidation (LPO) assay*

For the LPO assay, thiobarbituric acid reactive species (TBARS) were used according to the method described previously by Astaneie *et al*. 2005 (19).

All data obtained were expressed as Mean ± standard error mean (SEM) and compared using one way analysis of variance (ANOVA). The *p *<0.05 was considered statistically significant.

## Results and Discussion

From the underground parts of* A. tenuifolia*, two compounds **1** and **2** (Figure 1) were isolated and identified as β-sitosterol [**1**] and prangol [**2**] based on the spectroscopic spectra (^1^H-NMR, ^13^C-NMR and HSQC) compared to the known standard compounds which reported in the literature (20-22). ^1^H- and ^13^C-NMR data of the compounds **2** in CDCl_3 _is reported in Table 1. To confirm the identification of the compounds **1** and **2**, the isolated compounds were injected to MPLC again in the presence of their standard compounds that had already been identified from other plants and reported in the literatures.

The results of the AAI assay (Table 2) exhibited a dose-dependent inhibition for purifying compounds, while the inhibiting percentage decreased by increasing the concentration of hexane extract. The highest inhibitory activity of hexane extract of the plant was found as 50.2 ± 1.0 (%) at 10 mg/mL. It could be due to the conformational alteration of the enzyme by increasing the concentration of the compounds bonded (23). Acarbose was employed as a positive control and showed inhibitory activity at all used concentrations (10, 15, 20 and 25 mg/mL). However, its highest inhibiting percentage (82.1 ± 2.3) was at a concentration of 25 mg/mL. The tested compound β-sitosterol showed inhibitory activity with IC_50_ value of 18.9 ± 2.1 mg/mL. But prangol [**2**] showed almost no enzyme inhibitory activity in the esting concentrations. The results of viability assay are shown in Figure 2. The cell viability assay showed that just the highest concentrations of the plant extract (1000 and 10000 μg/mL) exhibited increased mitochondrial activity significantly higher than the control group (129.2 ± 8.49 and 177.3 ± 13.0%, respectively). The results of insulin secretion (Figure 3) showed that islets treated with 10000 μg/mL of the plant extract exhibited significant increase of insulin secretion (113.3 ± 2.0 and 122.5 ± 2.8%) at both glucose levels of stimulation (2.8 and 16.7 mM, respectively). Also, a significant increase of secretion was observed at 100 µg/mL of herbal treatment with glucose stimulation at 16.7 mM but not at 2.6 mM. In order to measure the cellular antioxidant activity, although, the percentage of intracellular ROS generation was evaluated and the results (Figure 4) showed that there was no significant decrease in concentrations of 1, 100, and 1000 μg/mL of plant extract, ROS generation (91.7 ± 4.4 and 87.7 ± 3.9%) were significantly reduced at 10 and 10000 µg/mL concentrations of the plant extract, respectively. Following the results, previous studies show that different species of *Achillea* have antioxidant effects on various organs such as brain and liver (24, 25). There is a relation between reduction of cellular oxygen species by plant extract and protecting pancreatic beta cells against apoptosis and necrosis via ERK1/2- and PI3K/Akt-mediated heme oxygenase-1 (HO-1) up-regulation (26). Furthermore, caspase-3 and -9 activities were determined by a colorimetric assay (Figure5) and it was revealed that only the highest concentration (10000 µg/mL) of herbal extract could significantly decrease the enzyme activity for both caspase-3 and -9 (64.7 ± 2.2 and 54.7 ± 2.8%, respectively), while a decrease in the enzyme activities could also be observed at 10 µg/L of the plant extract treatment. Measurement of cellular LPO also showed a similar result with other cellular antioxidant analysis (Figure 6), in which a significant decrease of LPO can be observed at 10000 µg/mL of the plant extract (15.3 ± 0.1% µg/mL) compared to control group, while a significant increase was determined at 10 µg/mL concentration of the plant extract. Finally, FRAP assay confirmed that the higher concentrations of *A. tenuifolia* root extract (1000 and 10000 µg/mL) was able to enhance the capacity of cellular antioxidants (68.2 ± 9.5 and 268.25 ± 12.2 mM, respectively) compared to control group (Figure 7). The results of previous studies showed that antioxidants have the ability to improve defense mechanisms of islet cells to oxidative stress through the direct antioxidant effect or modulation of beta cell apoptosis initiation and signaling (27). Therefore, natural antioxidants like medicinal plants and their active constituents can be applied in the management of diabetes. Our results are consistent with previous observations suggesting that plant extracts directly stimulate insulin secretion or improve islet cellꞌs defense in oxidative stress (28).

## Conclusion

In conclusion, *A. tenuifolia *root extract is able to enhance the function of beta-cells with increasing secretion of insulin and enhancement of their viability, as well as reducing the levels of ROS and LPO especially in higher concentrations. However, we could not test the isolated compounds except for AAI assay due to the minor amounts of them in a small portion of the plant roots available. It seems that β-sitosterol plays an important role in anti-diabetic activity of the plant roots partially via AAI activity. But prangol, another isolated furanocoumarinic compound, does not show AAI activity, although it may be effective via other mechanisms of action. Further investigations of the latter compound are recommended.
